# Becoming a Different Person: Living with Hepatic Encephalopathy as a Condition in Everyday Life—A Qualitative Explorative Study

**DOI:** 10.3390/healthcare14070874

**Published:** 2026-03-28

**Authors:** Marie Louise S. Hamberg, Rikke Parsberg Werge, Susanne Vahr Lauridsen, Thora Skodshøj Thomsen

**Affiliations:** 1 Department of Gastroenterology, Herlev Gentofte Hospital, 2730 Herlev, Denmark; susanne.vahr.lauridsen.02@regionh.dk; 2 Department of Gastroenterology, Amager Hvidovre Hospital, 2650 Hvidovre, Denmark; 3 Department of Surgery and Urology, Herlev Gentofte Hospital, 2730 Herlev, Denmark; 4 WHO-CC Parker Institute, 2000 Frederiksberg, Denmark; 5 Research Unity of Ophtalmology, Zealand University Hospital, 4000 Roskilde, Denmark; thst@regionsjaelland.dk; 6 Institute for Regional Health Research (IRS), University of Southern Denmark, 5230 Odense M, Denmark

**Keywords:** hepatic encephalopathy, liver cirrhosis, chronic liver disease, qualitative research, patient experience, identity, stigma, vulnerability, interpretive description

## Abstract

**Highlights:**

**What are the main findings?**

- Becoming a different person is the overall experience when living with hepatic encephalopathy. This fundamental change in identity has mental, social and physical implications for each individual patient.
- Vulnerability and (self-)stigmatization are inherent characteristics for this group of patients. A continuous awareness of this risk is crucial for anyone involved in treating and caring for this patient group.

**What are the implications of the main findings?**

- The population of patients living with liver cirrhosis is growing worldwide. A large proportion of these patients will experience hepatic encephalopathy. Hence, it is important to comprehend the lived experiences of this patient group. This study contributes to a sparsely studied area.
- Findings highlight the need for individualized and unconventional care, early and repeated education, and elaboration of hepatic encephalopathy (HE)-specific consultations. Continuously addressing the related stigma and vulnerability and supporting relatives are essential to improving quality of life for patients with cirrhosis.

**Abstract:**

Background/Objectives: Patients with liver cirrhosis experience a high symptom burden and low Health-Related Quality of Life (HR-QoL). Hepatic encephalopathy (HE) occurs in 75% of patients with cirrhosis but is sparsely described from the patient’s perspective. Due to recurrent cognitive impairment, a marginalized diagnosis, and a healthcare discourse emphasizing involvement and self-responsibility, these patients appear vulnerable when navigating a complex healthcare system. This study aims to explore how patients with chronic liver disease experience living with HE as a recurring condition, and how these patients are met by healthcare professionals (HCPs). Methods: Eight semi-structured interviews were conducted with four patients and four HCPs. Data were analyzed thematically following Braun and Clarke’s six-step analysis within the framework of Interpretive Description. The study was reported according to COREQ Guidelines. Results: The overarching theme “Becoming a different person” captured the profound identity changes experienced by patients. Three main themes emerged: 1. change and loss—in identity and self-understanding, in relationships, in relation to losing control, and in relation to experiencing isolation; 2. new paths—mental and practical alternative strategies; 3. HE in clinical encounters—requiring empathy, flexibility, and continuity. Stigma related to cirrhosis and its association with alcohol further intensified patients’ vulnerability. Conclusions: HE is experienced as a transformative and isolating condition, deeply affecting patients’ autonomy and social roles through vulnerability. The clinical encounter is shaped by the cognitive impairment due to HE, requiring tailored and sensitive care.

## 1. Introduction

This paper concerns the patient perspective on living with end-stage liver disease (cirrhosis), particularly those experiencing the symptom hepatic encephalopathy (HE). Furthermore, the patient perspective will be supplemented by that of healthcare professionals working with this patient group.

Liver cirrhosis is considered the irreversible end stage of chronic liver disease. It is the result of a slow conversion of the liver from functional liver tissue to inactive connective tissue. This leads to reduced liver function [1]. Cirrhosis denotes a complex of symptoms, e.g., esophageal variceal bleeding, fluid retention/ascites, sarcopenia, fatigue, weakness, and HE, the symptoms potentially increasing in number, severity and intensity as the disease progresses [2,3,4]. Thus, patients with liver cirrhosis live with a high burden of symptoms and as a result a well-documented low Health-Related Quality of Life (HR-QoL) [5,6].

Cirrhosis is often attributed to an overuse of alcohol but can also be caused by viruses, toxicity, autoimmunity, and genetic disorders [1,7]. Also, metabolic dysfunction and obesity is an increasing cause of cirrhosis [7]. In particular, metabolic liver disease is predicted to continue to increase for the coming decades [8]. Thus, cirrhosis poses an increasing health challenge worldwide [9,10], but already the global burden of cirrhosis is considered substantial [11]. In Denmark, the prevalence of cirrhosis is approximately 14,000 people, the incidence being 1700 per year [1]. Approximately, 75% of all patients diagnosed with cirrhosis will, during their disease trajectory, develop HE, ranging from minimal/covert to overt HE; of these patients, 30–40% will experience HE grade 2 or more [3,4] (see Table 1). HE is the cirrhosis symptom which most frequently causes admission and readmission to hospital. Likewise, HE deeply affects HR-QoL for both patients and their nearest [5], and despite a well-documented quantitative low HR-QoL, a systematic literature search shows that HE is qualitatively sparsely described from a patient perspective.

### Background on HE

HE is a reversible, recurrent cognitive impairment characterized by non-specific neurological and mental manifestations affecting both consciousness and personality [1,12]. Clinically, HE is classified according to the West Haven Criteria as minimal HE (MHE) and four stages. MHE is a discrete, not immediately clinically apparent change in cognition and psychomotor functions. In grade 1, patients have mild changes in behavior and personality, often only noted by relatives. Patients may present with changed circadian rhythm, inattention and fatigue. Grade 2 is characterized by disorientation, psychomotor slowing, and impaired performance of everyday tasks, and may include inappropriate behavior, altered handwriting and speech, and asterixis. In grade 3, patients are markedly confused and somnolent, and frequently difficult to wake up, whereas grade 4 represents coma with unresponsiveness and a need for airway protection and ventilatory support [1,4].

Many studies among patients with cirrhosis identify recurrent HE as the major contributor to reduced HR-QoL, defined as individuals’ perceived physical and mental health over time [5,13]. HE is often described as the most feared symptom among patients with liver cirrhosis [14], and evidence shows that it leads to impairment in both practical skills and communicating abilities including expressive and receptive functions [12]. Thus, patients with cirrhosis and HE live their everyday life with the condition that even minor physiological changes can result in major cognitive impairment involving both consciousness and personality.

In Denmark, as in many other countries, the healthcare system emphasizes the importance of equality in health through encouraging, e.g., patient involvement and shared decision-making [15,16]. However, these aims are challenged in patients with HE due to cognitive impairment, which affects their ability to engage in care and manage everyday life. As a result, these patients may experience increased vulnerability in their interactions with the healthcare system, where expectations for participation can exceed their functional capacity [17,18,19].

Despite these major consequences for this patient group there remains a lack of research exploring the lived, subjective experience of HE as a key manifestation of cirrhosis, regardless of etiology. This lack is substantiated through numerous structured searches of the scientific literature. The gap is also consistently mentioned in the literature [3,5,12].

Against this background, the aim of this study was to explore patients’ lived experiences with HE in everyday life and how this bilaterally affects the clinical encounter between patients with HE and HCPs. This aim gave rise to the following research question: How does a patient with chronic liver disease experience living with HE as a recurring condition, and how are these patients met by the HCPs in clinical practice, seen from the perspective of both the patients and the HCPs?

## 2. Methods

### 2.1. Design

The study was based on the qualitative methodology Interpretive Description (ID) according to Sally Thorne [20]. This was chosen as ID is a pragmatic and praxis-oriented framework focusing on developing knowledge for practice. Since the research question in the present study rises from clinical practice and demands to be explored in clinical practice, ID as an undogmatic approach offers flexibility, meaningfulness and relevance for both researchers and participants. It also helps to keep focus on a practice-oriented development of new knowledge in the explored field in the interest of the people examined.

ID does not present a fixed and controlled procedure but describes five generic elements constituting the research process [20,21]:

1. Scaffolding—refers to the explication of the position of the researcher(s), the scientific theoretical point of view and a review of existing literature of relevance. In this study, the researchers’ preconceptions were made visible through conceptual frameworks according to JA Maxwell [22];
2. Design;
3. Data construction;
4. Data analysis;
5. Discussion of the implication of the findings for clinical practice.

In appreciation of qualitative research being a creative, iterative, and complex process, we used an *audit trail* to create transparency during the entire research process. This way we documented ideas, reflections, and the background for decisions and opt-outs through informal written formats [20].

The study was reported according to COREQ Guidelines [23].

### 2.2. Recruitment and Sampling of Participants

Eligible patients with recent HE episodes were consecutively identified and purposively recruited over two months; of seven approached, four were included. Three were excluded—one because of lack of recognition of cirrhosis as the cause of the recurrent cognitive deficit and two because of severe cognitive impairment (HE grade ≥ 2) at the time of recruitment.

A purposive sample of nurses and doctors from inpatient and outpatient hospital settings was recruited to ensure diverse perspectives. Healthcare professionals were informed about the study by mail. The first four colleagues asked (two nurses and two doctors) agreed to participate, representing two to 15 years of experience with the patient group.

All included patients had retired due to age or diagnosis and were literately educated for a minimum of 4 years. Cirrhosis diagnosis was due to ALD (alcoholic liver disease) or MASLD (metabolic-associated steatotic liver disease) according to EASL (European Association for the Study of the Liver) terminology [24], with participant characteristics shown in Table 2.

### 2.3. Inclusion and Exclusion Criteria

Inclusion criteria, patients: patients age ≥ 18 years, the ability to speak and understand Danish, and at least one admission with verified HE within the last 8–10 weeks, along with self-recognition of cirrhosis as the cause of their cognitive impairment.

Exclusion criteria, patients: Other potential causes of cognitive impairment, patients with cirrhosis without HE, patients who were too cognitively impaired in general or at the time of the interview (HE grade ≥2), as well as patients who did not recognize cirrhosis as the cause of their cognitive impairment.

Inclusion criteria, HCP: the ability to speak and understand Danish and a minimum of 2 years of professional experience in treating patients with end-stage liver disease and HE.

Exclusion criteria, HCP: less than 2 years of experience in hepatology.

### 2.4. Data Collection and Settings

Data was collected between February and March 2023 at a university hospital in the capital area of Copenhagen. We conducted 8 semi-structured interviews inspired by Tanggaard & Brinkmann [25], four with patients and four with HCPs (Table 2). Data collectors were experienced nurses and master students with sparse research experience.

Patients were initially contacted in relation to a recent admission. After consent, they chose the location for the interview and were encouraged to let the interview take place in their own home to facilitate a sense of security but also to enhance and produce everyday experiences related to living with HE as a condition in everyday life [26]. The severity of HE at the time of interview was clinically assessed without the use of psychometric tests. Both first authors were present at the four interviews, taking turns being limited to observe and take care of the sound recording. This gave the opportunity to optimize the collection of data, both linguistic and sensory [26]. The semi-structured interviews were performed over a period of 10 days and lasted 45–70 min.

All four HCPs were interviewed at the hospital according to their own choice; only one author was present and the interviews lasted 35–45 min.

Two interview guides were developed with inspiration from Tanggaard & Brinkmann [25] and Green & Thorogood [26], one for patient interviews and one for interviews with HCPs. The interview guide for HCPs was created after all patient interviews were conducted, allowing these to inspire the guide. Both interview guides consisted of open-ended questions, each with follow-up and probing questions to ensure clarity and depth in the participant’s answers [26] and can be found in the Supplementary Materials Section.

Both first authors were involved in recruitment and interviewing. All interviews were performed without any previous contact or relationship between the interviewer and the patient or colleague. They were conducted face-to-face, recorded, and transcribed verbatim by both first authors for later thematic analysis.

### 2.5. Data Analysis

A hermeneutic approach was applied throughout data analysis. Guided by the research question, the data set was decontextualized and recontextualized in order to move from specific individual experiences toward a representation of more general descriptions of the meaning of these experiences.

ID states that with mere description only half of the analytic work is done, thus emphasizing the intertwined and sometimes messy process of interpretation as a cornerstone [20]. In the absence of a specific procedure within ID, the analysis process was inspired by *Thematic Analysis* described by Braun and Clarke [27] as recommended by Thorne [20]. The findings were thought, spoken, and written out from across data by both first authors. The 6 steps of analysis according to Braun and Clarke are familiarization of data, generation of codes, combining codes into themes, reviewing themes, determine significance of themes, and reporting of findings.

**Figure 1:**
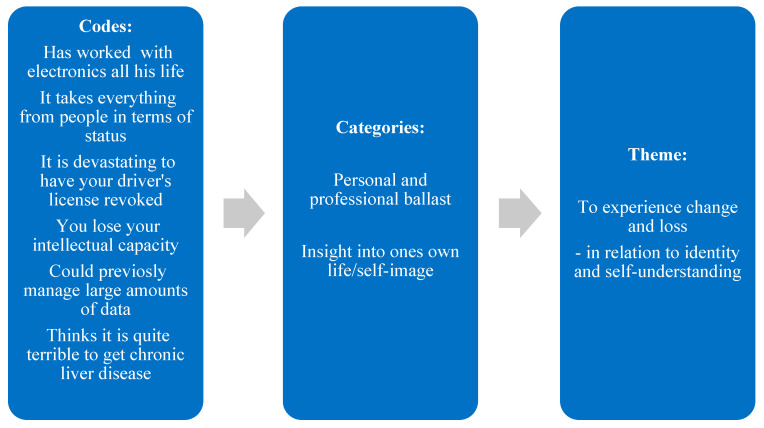
Example of analysis process.

The analysis process was initiated with careful listening and transcription of the recorded interviews, thorough reading to familiarize with the content and a description of content in semantic codes. The codes were then inductively gathered in categories, moving back and forth in the entire data set. Both codes and categories were then again compared, to establish themes that were both clear and distinct; each theme should be internally coherent in terms of the codes and categories covered. At the same time no two themes should overlap. Finally, the themes were experimentally combined and rated as superior or inferior, to ensure relevance and meaningfulness according to the research question. In this process, the themes went from being expressed in terms to pairs of terms and then combined with a verb, which emphasizes a practice perspective. Thus, the analysis brought us from descriptive codes to unifying iterative categories towards a coherent, meaningful description and interpretation into themes describing the general commonalities found across data. These were overarching and subordinate, as well as mutually related. The analysis was completed manually, with no software used. The process of analysis is shown in Figure 1.

### 2.6. Ethics

Written consent to conduct the study was obtained from the head of department. Patients and HCPs were informed about voluntariness, anonymity, and the opportunity to withdraw consent at any time according to national legal standards, both orally and in writing [28]. The study was registered with the Data Protection Authority on 17 January 2023. Under Danish law no approval from an Ethics Committee was necessary. All data was anonymized and digitally stored according to legal standards for data protection [29]. The study overall complies with the ethical research standards as described in the Helsinki declaration [30].

Any study of patient groups with cognitive deficits, e.g., recurrent HE, will demand a high degree of ethical considerations and delicacy since research basics, e.g., informed consent, voluntariness, and data validity, could be questioned [31]. At the same time, research among these vulnerable patients is often considered to be slow and cumbersome and is therefore sparse [31]. The Helsinki declaration [30] addresses this ethical issue with a demand to listen to those who rarely speak up in terms of specifically giving research access to vulnerable and underrepresented patient groups. The declaration states that research among vulnerable patient groups is legitimate if the same knowledge cannot be produced in any other way and with the obligation that the patients in the future must profit from the knowledge produced [30]. Therefore, in this study patients with HE were recruited if they could give informed consent (they were asked several times) as well as having a relevant conversation.

## 3. Results

**Figure 2:**
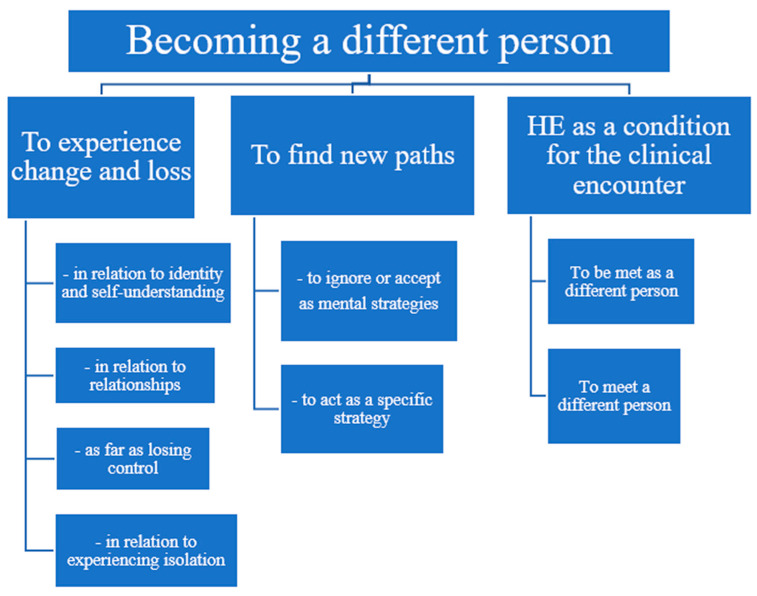
Findings: one overarching theme, 3 main themes and 8 subthemes describing patients’ life with HE.

Since the research question calls for two contemporaneous and intertwined perspectives, the patient’s and the HCPs’, the findings will be presented likewise. Below, the overall finding, three themes and eight sub-themes (Figure 2), as well as their mutual relationships, will be further unfolded and described.

In quotations, the anonymized participants will be referred to as shown in Table 2.

### 3.1. Becoming a Different Person

The overall finding across this data set was a profound experience of becoming a different person, both physically and mentally, when experiencing HE. This was identified as the overarching theme and expressed by one patient as follows:

P3: *“It was like two different worlds … it is the best description I can give, I was in one world and they* (people around) *were in another … I couldn’t reach out to the world. The world couldn’t hear me.”*

From the point of view of the HCPs it was described as “*a global impairment*” (HCP3). The patients with HE are found to lose independence and need support to be both a patient and even a person:

HCP3: *“The patients simply lose a part of themselves, they are not as much part of their own lives as they used to be—clearly, that is an existential pain.”*

It became visible both explicitly and from within the patients’ stories that this “different person” is a vulnerable person.

### 3.2. To Experience Change and Loss

The patients described how changes and losses linked to HE manifest in different domains. E.g., their sense of self was reduced—their self-image no longer matched who they used to be or wanted to be because HE was both physically and mentally decisive. This was seen to various degrees, from existential worries over pervasive changes in everyday life to minor obstacles and adjustments. The HCPs saw this when meeting the patients as well:

HCP3: “*It’s the loss of oneself—because that is what one loses* (with HE).”

- In relation to identity and self-understanding:

In different ways, patients described a former life characterized by structure, responsibilities, and commitment—personal characteristics that were compromised with HE:

P4: “*It’s a tremendous mental loss, and it was very uncomfortable … well, when you’re an intellectual person capable of having a lot on your plate* (crying) *…—it’s very uncomfortable*.” Also, between HE episodes patients had to limit social activities in order to prevent another HE episode because of overexertion. E.g., the role as a grandparent was dosed and visits were planned and shortened.

Loss was also present when patients were no longer able to drive their car—that sense of dependence was hard for patients to accept, which was also acknowledged by HCPs: *“If you have your license taken, it’s simply too much, they can’t take it, and they don’t feel themselves, that they can’t control it.”* (HCP1).

In very specific ways, HCPs experienced their patients’ changed identity, as it is often necessary to involve a third party to support the consultations because of the reduced cognitive and communicative capacity that HE entails. The loss was expressed from both perspectives:

P4: *“It’s a huge, huge loss of authority in one’s own life, that’s what happens.”*

HCP3: *“You take everything away from these people, right—also in terms of loss of status … I think we forget that it is … how devastating it is to lose one’s mind like that.”*

- In relation to relationships:

The participants all had grown-up children—in these relationships they experienced a shift in roles. Several had a wish to still take care of their children, fighting for them more than for themselves. One participant was painfully aware that it is a great fright to experience HE in a close relative. The participant had at one point established a book with drawings and descriptions from HE episodes, made to process the experiences:

P3: *“… but I think I got rid of the book because I felt it would be too much for the children to find … if I’m suddenly not here any longer and they should find a book like that, I wouldn’t like that.”*

Moreover, death as an outcome becomes present in the relations between patients and relatives. The reversibility of HE if the symptom is appropriately treated is not present knowledge—this was reflected in the following quote:

*“I know that the children came to say their last goodbyes to me the last time—when I was in a coma”* (P3).

Likewise, the HCPs acknowledged how relations were altered for their patients when HE was present. E.g., this means relatives that are “*squeezed, overwhelmed and in crises*” (HCP3). When HE limits the patient, the relatives often fill in the gap—the relatives are met as “part of the patient”. This altered distribution of responsibility often happens imperceptibly and out of necessity:

*“They* (the patients) *are of course much, much more vulnerable if they don’t have a spouse, because then they can just sit at home and nothing happens, neither with food, exercise or anything, and they can’t either get themselves admitted if they see worsening in symptoms because they just don’t recognize it.”* (HCP1).

Thus, patients with HE experienced a shift in roles but still felt a need to protect grown-up children despite their own vulnerability. Relatives felt overwhelmed when taking on additional responsibilities.

- Regarding losing control:

Across data, losing control appeared to come in both episodic and continuous forms. The continuous loss of control mainly refers to the above description of an altered sense of self and identity followed by diagnosed HE. The episodic loss of control is linked to the intermittent character of the symptom. Patients described how everyday life skills such as taking off or putting on clothes or using a phone suddenly became impossible. It was described as a universal feeling of a head that did not cooperate and not being able to figure anything out.

Several patients described having behaved in ways that violate common norms and unwritten conventions of how to express illness. Some described being distrustful and skeptical of those normally trusted. Others described having slept on and crawled around the hospital floor or, as this patient told:

P4: *“… and then I sat on a dining room chair and then I peed … and that’s extremely uncomfortable, right, that loss of control”.*

A common feature of the patients’ stories was that they did not remember what happened during an HE episode but were told by relatives or staff afterwards. This emphasizes a duality in the loss of control: HE causes patients to act unusual and transcendent and, at the same time, they are not able to remember and reconstruct the course of events without help from others. This points to an ultimate sense of loss of control when HE manifests.

P2: *“Done something odd? I must have! I can’t remember right now* (laughing), *I must have repressed that! … I haven’t noticed myself—my surroundings may have, but I haven’t!”*

The loss of control was experienced by the HCPs as adding to the vulnerability of these patients. This has a practical dimension as far as being able to maneuver the healthcare system, which requires a high degree of self-reliance. At the same time, the existential dimension is very present:

HCP4: *“There is also this uncertainty about when it will hit next, it’s … you know, now I have had this* (HE) *once and it will most likely come again at some point—when and where and will I be able to read the signs, because it’s often others who discover it … I think it must be extremely uncomfortable to walk around with that knowledge, right?”*

Both continuous and episodic loss of control affected the patients’ daily skills and behavior. The duality of losing control coupled with the inability to remember the HE episodes adds to both vulnerability and existential anxiety—it becomes challenging to navigate life.

- In relation to experiencing isolation:

Even though half of the participants were cohabiting and three of four described a close relation to friends and family, social isolation was a pervasive and prominent finding in the description of HE experiences across the data set. The sense of isolation was present both during and in between episodes of HE. Physically, isolation was a matter of limited range of action. Due to cirrhosis, patients experience weakness, fatigue, and impaired balance, and several of the participants had their driving license revoked. They also described having to limit outgoing activities, tours and travels as well as housing guests in order to prevent new episodes:

P4: *“… and then there must be days of no activity before I see people again … it can’t be too much and also I don’t go abroad anymore. We’ve been traveling a lot; my husband still wants to, and I tell him he can go, but I won’t.”*

Despite the patients not being able to reconstruct the specific course of events during an HE episode they still described a sense of mental isolation, of being “somewhere else”, and a sense of parallelism, not being able to communicate with those around, as illustrated in the following quotes:

P2: *“It’s like being in one world, so to speak, and looking into another world.”*

P3: *“It made sense to me … but it didn’t to them … I tried to get in contact with them* (those around) *to tell them what happened inside me and I got so mad, that they didn’t respond when I tried!”*

Thus, HE seems to put patients in a bubble, all alone and disconnected from surroundings. One patient expressed it as follows:

P4: *“I had a sort of mantra, I said to myself: You cannot be demented in only one day.”*

This was confirmed by the HCPs, as they experienced the patients as passive and retreating with HE.

### 3.3. To Find New Paths

Living with HE as a recurrent condition creates a need for reorientation in terms of both specific and mental ways of coping in life. The developed strategies can be more or less suitable and they can be used consciously or unconsciously. The strategies vary widely among individuals, reflecting differences in knowledge, acceptance, and personal adaptation to the diagnosis.

- To ignore or accept as mental strategies:

The extent to which the patients used factual knowledge about their liver diagnosis, and HE in particular, varied across data and showed no immediate pattern. Some used technical and academic terms such as “encephalopathy” while others had no specific term for HE but used words such as “fog” or “drowsiness” to describe an episode. Moreover, this different use was inconsistent across the participants, as one could show a high degree of knowledge and competence to act in specific areas of life and at the same time have no language for life with HE.

The cognitive impairment and lability caused by HE itself plays a role here. At the same time there seems to be an element of ignorance involved; e.g., one patient describes how HE was thought to originate from something totally different than liver cirrhosis, namely cancer or a heart condition.

The position of ignorance or distance was also recognized by the HCPs. The symptom HE not always being connected to liver failure by the patients was recognized by several colleagues, and one confirmed this:

HCP3: *“Yes, that can be both a denial or a misunderstanding … I think it’s part of making this history their own—it’s the thoughts one carries in private; one tries to reconcile with or understand, how the situation is; I believe, you also give yourself some explanations, and they can come from everywhere…”.*

Acceptance was also found to be present among the participants through both their language and their attitude when asked if it is problematic to have a liver disease:

P4: *“No, because that’s what a have!”*

Others used phrases such as “*that’s the way of life*” (P2) and expressed no worries:

P1: *“Right now I find I’m feeling perfect.”*

Patients thus developed mental strategies to cope and adapt to life with HE, ranging from ignorance to acceptance. To ignore and accept appeared as a convoluted attempt to succeed. The strategies reflected individual levels of knowledge and adaption, helping patients to navigate life despite cognitive impairment. Data seemed to show a proportionality between the knowledge and understanding patients possessed and the degree of acceptance they expressed.

- To take action as a specific strategy :

Taking action as a specific strategy to manage life with HE was expressed across data in how patients re-arranged themselves in everyday life. We found that every patient in individual ways would tread new paths to help themselves in useful and straight forward ways. Alternative ways were sought, e.g., the use of water bottles in the refrigerator that should be drank in full each day to avoid dehydration, or sticky notes to remind them about medication and appointments. Some brought previous competencies into new use:

P1: *“… and here it says* (in electronic calendar) *Lactulose, it’s 7 p.m., and I get a notification a few hours ahead, that makes me remember … I put everything in there!”*

The strategies were not necessarily tied to in-depth knowledge about prevention of HE but in any case, they seem useful to participants.

HCPs saw a major educational task in empowering patients to manage everyday life:

HCP3: *“A large part of the task we solve here, including the nurses in the out-patient clinic, is to approach what it means to live a life with a liver diagnosis—that way patients can adjust and be as self-determent as possible about their course of treatment, right?”*

### 3.4. HE as a Condition in Clinical Encounters

Across all interviews, HE was found to be a very peculiar symptom creating very special conditions for the clinical encounter, significantly different from that with other patient groups. Overall, patients expressed satisfaction with their contact with the healthcare system in terms of the symptom and diagnosis in question, and HCPs described both empathy and challenges when meeting these patients, whom they perceived as particularly vulnerable.

- To be met as a different person :

HE seemed to be an underlying ubiquitous premise, and some patients explicitly described their need of being cared for in special and sometimes unconventional ways. E.g., we heard patients wishing to sleep on the floor in the ward (which was granted); others described being distrustful, agitated and not wanting to receive the help they needed—all because of the cognitive impairment by HE. This also stresses the symptoms’ intermittent character: during one admission patients would go from angrily refusing to receive help to maybe the next morning being themselves again, hearing about what they have said and done while being “in another world”.

Several patients stressed the importance of continuity as it provides a sense of security. It is important to be recognized:

P4: *“I like to see the same person, because then you don’t have to start from Adam and Eve again.”*

This was confirmed but also challenged, as being well known means frequent admissions:

P3: *“Then he* (the nurse) *said: ‘Hi* (patient’s name)*’—not very flattering, but he did.”*

Overall, patients expressed that they had received useful help and felt welcomed and at home:

P2: *“They just burst in and ask* (smiles) *…—it’s very straight forward, not formal, and solemn … I like that.”*

However, it was also told how HCPs could represent prejudices and ideas of self-infliction, on the basis of the conception of the perceived close relationship between cirrhosis and alcohol; this had great significance for the patients:

P4: *“… then I said ‘I’m not … it’s not because of alcohol’ and the staff responded ‘Well right, they all say so’. ‘But it’s not’ … and then I said to the doctor, that I wanted it to be stated in my file that it wasn’t because of alcohol and then I could just refer to that … but other than that, they were nice.”*

Finally, it should be mentioned how patients experienced the clinical settings as a sanctuary where the concerns for relatives can be disregarded for a while. In that regard, HCPs play a central role in patients’ consciousness:

P3: *“I do need a safe space … they* (HCPs) *cannot disclose anything!”*

This is considered a significant finding as it represents an explicit patient need which is opposed to the essential importance of involving close relatives in the clinical encounter in order to make the information given in the hospital understood and remembered: a close relative is in practice often considered the only person with access to a patient under HE influence, a deputy to communicate through, “*a proxy for the patient sitting next to him/her*” (HCP3). But still, the patient has a need for privacy.

- To meet a different person :

Across all interviews with HCPs it was evident that they empathized with and cared for this patient group in a special way. It was explicitly stated, both in choice of words and tone of voice, that they know they do not necessarily share this bond with all colleagues across specialties:

HCP4: *“I believe that doctors ending up in this specialty have some kind of, I don’t know if you can call it love, for these patients, at least not resistance as other places. Some colleagues definitely show that maybe in other specialties*.”

The HCPs were fully aware of meeting a patient with very significant challenges who temporarily has become “a different person”. They described = professional, structural, and organizational challenges in the clinical encounter, ranging from minor communication difficulties over handling and shielding an outgoing and aggressive person to treating someone in a deep coma:

HCP4: *“It can become troublesome because the patients often get drowsy, but they also sometimes make trouble—it’s primarily the nurses being responsible for them in the ward; they can become really burdensome, that takes up a lot of capacity*.”

In every clinical encounter, HCPs must tune into the patients’ cognitive capacity to communicate. This can be done in basic ways by, e.g., asking for today’s date, personal ID number, or the name of the current Prime Minister:

HCP1: *“It’s sometimes hard, because you are left with the feeling of not knowing whether any of this got through, or if what I just said made any sense …*”

Several expressed a special obligation to communicate clearly and honestly, protecting the patients’ integrity as they expectedly cannot do that themselves. They also stressed the importance of being ready to take unconventional steps in the best interest of patients. Because the patients intermittently act in uncommon ways, this should be answered with untraditional solutions when appropriate, cf., allowing the patient to sleep on the floor, ignoring comfort and hygiene considerations.

One HCP summarized that *“working with HE means to articulate a necessary and more or less temporary loss of co-determination*”, which calls for willingness to act outside norms and regulations in the patients’ best interest. “*You sometimes have to take some detours*.” (HCP1).

Across interviews it was mentioned how HE must be the most difficult cirrhosis symptom to handle—it is easy to relate to because it is a common human experience how important our consciousness and cognition are to our identity and self-understanding. This was put into words as a new insight at the end of an interview:

HCP4: “*But of course, it is a very difficult message to get: ‘because of your liver your brain is affected; we can help you, but it will most likely reoccur at some point’ … I don’t know if I think about this enough, and you really must; the first time you give this message to someone, it’s really something that needs to be addressed*.”

### 3.5. The Impact of Alcohol and the Cirrhosis Diagnosis

This study aimed to investigate the experience of HE as a symptom of cirrhosis only. However, during the interviews, the diagnosis of cirrhosis and its frequent association with alcohol emerged consistently. Participants expressed a sense of self-stigma and perceived self-infliction which appeared to contribute to their overall vulnerability. These perceptions were relevant to how participants described the changes in their sense of self associated with HE. This was noted even among those without a history of alcohol use:

P4: *“And the cirrhosis I have, it’s NOT alcohol related—and to me it* (the etiology) *does matter, because I never have been drinking …”*

P3: *“You wouldn’t say that to a person with a broken leg, that ‘you’ve brought yourself into that situation’ … and that shows how difficult it is, …., it’s your own fault. … You get a feeling of belonging to a problematic group of citizens …”*

The finding of self-stigma was summarized in the following quotation: “*It all depends on whether you accept liver cirrhosis to be …—a disease …?”* (P3).

## 4. Discussion

The main finding in this study shows how the societal and public perception of the diagnosis of liver cirrhosis and its relation to alcohol is inseparable from the experiences of the patients. Thus, HE patients’ experience of becoming “a different person” is not only linked to the symptom itself and the vast implications HE has for everyday life. It is also linked to the diagnosis, to alcohol, and to the associated stigma. This is found in the literature, too, where Mikkelsen et al. [14] describe how patients with alcoholic cirrhosis experience being looked down upon, both in society and among HCPs. The stigmatization affects patients’ feelings of shame and isolation, reinforced by the general perception of cirrhosis as self-inflicted [12,32]. This points to the assumption that several societal determinants play a significant role when characterizing the “different person” that patients with HE become.

The patients experienced various forms of changes and losses, including loss of self-understanding/identity, altered relationships, and isolation. The study’s findings reflect Grønkjær et al. [3,12], who describe loss of identity and control and social isolation—“multiple losses”. Although anxiety was not directly mentioned by the patients, they experienced existential insecurity due to the unpredictability of HE. In Grønkjær et al. [3,12] and Fabrellas et al. [5], anxiety during and after HE episodes was described as a persistent feeling of insecurity and fear of future episodes, which adds to feeling helpless and vulnerable. Mikkelsen et al. [14] found that patients feared HE as a frightening complication, this added to a constant feeling of anxiety, as the symptom is recurrent.

The patients’ identity was deeply affected by both HE and the diagnosis of liver cirrhosis, which is also seen in the studies by Mikkelsen et al. [14] and Grønkjær et al. [12]. Dependence on others, often perceived as threatening to identity, was highlighted, especially in relation to daily activities such as driving and relationships with relatives. Fabrellas et al. [5] compare the dependence of HE patients to that of dementia patients, which was expressed by one of the participants in this study, too.

Mental and physical isolation was highlighted by the patients as a consequence of the unpredictability of HE and the physical limitations caused by liver cirrhosis. The choice between participating in social activities or staying home was often influenced by this uncertainty, which increased isolation and loss of control. Studies by Hjorth et al. [32] and Fabrellas et al. [5] confirm how unpredictability and the use of medications, such as laxatives, limit the patients’ social lives.

When patients lose the ability to control both their physical and mental state during a HE episode and become dependent on others to restore their memory, it was described as a double loss of control. This is also described by Grønkjær et al. [3] under the theme “multiple losses,” where the loss of control is linked to the experience of dependence on others. This further contributes to the patients’ vulnerability and sense of dependence, which is a recurring feeling in both this study and the existing literature.

Ignorance and acceptance appear as an intertwined mental attempt to adapt to a life with HE as a condition, where ‘taking action’ is also a strategy. The empirical data shows that patients often lack knowledge about HE or cirrhosis, which can be due to either deliberate ignorance or memory problems caused by HE. The lack of understanding of the diagnosis is also confirmed in the literature, including Grønkjær et al. [3] and Ismond et al. [13]. Coping strategies and acceptance of a new life situation are seen across both empirical data and research, where patients adjust their daily lives and seek information to manage the symptoms. Patient education and regular HE consultations are highlighted as important means to strengthen patients’ self-care and adaptation, as recommended by Ismond et al. [13].

There is a need for early and thorough information about HE so that patients can understand the unpredictable nature of their disease. It is important for HCPs to adapt their approach and sometimes use unconventional approaches in their care and treatment, as described by Hjorth et al. [33]. This is supported by Fabrellas [5], who describes the first HE episode to be particularly stressful because the patients have not been prepared for it and therefore find it very violent.

Patients value continuity in their encounters with HCP, which contributes to their sense of security and recognition, as supported by Hjorth et al. [33]. Although most describe positive experiences, a lack of information about the disease and treatment is also mentioned, which may be due to cognitive challenges caused by HE. HCPs are crucial sources of this information, and it is important to provide it when patients are ready and able to receive it, preferably in the beginning of the course of treatment. In the literature, no specific time point is mentioned as the right one regarding information given to patients about HE as a symptom of cirrhosis. This will therefore be a matter of ongoing assessment in the clinical encounters, also including the needs of relatives and the conscious use of repetition as a communicative tool.

Several HCPs express that they find HE to be the worst complication of cirrhosis due to its cognitive impact and the vulnerability it causes. The literature supports that HE is the most disabling complication of cirrhosis, being ultimately the most important determinant of the low HR-QoL in patients with cirrhosis [13]. Grønkjær et al. [3] describe HE as the most disabling symptom of cirrhosis, affecting both patients and their relatives, and in Fabrellas et al. [5], patients expressed in their own words that they would prefer any other cirrhosis symptom over HE.

Thus, the findings of this study are found to be influenced by both external and implicit determinants. It is not just about the burden of consequences for everyday life that the symptom HE entails. It is also about the reputation and stigma associated with the diagnosis of cirrhosis, the omnipresence of alcohol as a factor, and the stigmatization these determinants bring about.

## 5. Strengths and Limitations

There are both strengths and limitations to this study. Our recruiting strategy, which did not include medical assessment of the patients’ cognitive state or the commonly used psychometric tests for HE, might have resulted in some participants having MHE or HE grade 1 at the time of the interview. Assessment and tests were opted out as they were found to potentially disrupt the study focus and compromise trust and immediacy in the interview. ID emphasizes the importance of a diplomatic and respectful rendering of findings [20]. The first authors therefore took on a special obligation of attention, loyalty and transparency towards patients when interviewing, analyzing and reporting data in order to create and present the findings in the most trustworthy way possible [34]. Patients’ trust was answered with respect, recognition and confidentiality.

The four patients interviewed illustrated the continuum on which HE occurs, cf., West Haven (Table 1), thus representing breadth and variation in the appearance of the symptom. The findings have limited transferability and are not representative in terms of generalizability, but there is strength in the depth of the collected personal experiences that can thus create recognizability.

The research process was, from beginning to end, driven by the chosen explored field. This was done through the presentation of transparent reflections and ongoing decisions about approaches and methods. Furthermore, an audit trail was prepared to document the path and development of the research process. This was done to strengthen the validity of the study.

## 6. Relevance to Clinical Practice

Our study encourages reflections on both organizational and practical levels, advocating for a vulnerable patient group to ensure their voices are heard.

The ongoing clinical encounters act as the “opportunity space” for unconventional solutions, timely information and addressing stigma within the healthcare system as well as within society overall. This “opportunity space” is to be further explored and developed in local clinical settings.

1. We encourage development of “HE consultation courses”, considering content, frequency, timing, and participants, thus preparing patients and relatives for the potential symptom. Therapeutic communication [3] and the profitable sharing of HE stories among patients [13] can be integrated. Patient education, psychosocial support, and destigmatization are essentials to improve HR-QoL for patients with HE and cirrhosis [35].
2. The current national and international professional focus on early, basic palliation for patients with non-malignant disorders, including chronic liver disease, could be a relevant frame for clinical initiatives aimed at increasing the HR-QoL for patients with HE.
3. Future studies can approach data deductively with the concept of ‘vulnerability’ as a theoretical framework. This could highlight the significance of vulnerability due to both cognitive impairment (HE) and potential (self-)stigmatization.

## 7. Conclusions

Based on the sparse knowledge about experiences in patients with cirrhosis and HE, we aimed to uncover how HE is subjectively perceived, and what characterizes patients’ encounters with the healthcare system from both a patient and professional perspective. Additionally, we aimed to give a voice to a rarely heard, marginalized patient group.

In conclusion, our data show that HE can be described as a fundamental experience of becoming a different person through changes and loss of identity and control. This leads to the development of new paths in life through both mental and active strategies. The encounter between the patient and the healthcare system requires unconventional approaches in care and treatment as well as special attention to communication and relatives. As external determinants, the diagnosis of cirrhosis, especially due to its inherent relation to alcohol, carries a potential for stigma. This profoundly affects the patients’ experiences and adds to their vulnerability.

Thus, this study nuances existing knowledge and contributes to a clinical field with limited research. Our findings contribute to the understanding of the patients we work with as well as their context, enabling evidence-based practice focused on patients’ lived experience.

The overall synthesis of the findings shows both static conditions (the symptom, the diagnosis, alcohol, and stigmatization) as well as an “opportunity space” for the clinical encounter, which can be influenced and modified in local clinical practice. The underlying vulnerability can be met through the relationship between patients and HCPs (see Supplementary Materials for illustration).

## Figures and Tables

**Table 1:** West Haven Criteria [4].

Unimpaired		No Encephalopathy at All, No History of HE
**Minimal**	**Covert**	Psychometric or neuropsychological alterations of tests exploring psychomotor speed/executive functions or neurophysiological alterations without clinical evidence of mental change
**Grade I**		Trivial lack of awareness Euphoria or anxiety Shortened attention span Impairment of addition or subtraction
**Grade II**	**Overt**	Lethargy or apathy Disorientation for time Obvious personality change Inappropriate behavior
**Grade III**		Somnolence to semi-stupor Responsive to stimuli Confused Gross disorientation Bizarre behavior
**Grade IV**		Coma

**Table 2:** Participant characteristics. M = Male. F = Female.

Participant Patient	Gender (M/F)	Age	Living Alone (Y/N)	Time of Diagnosis	HE Grade at Time of Interview According to West Haven Criteria	Latest Admission
P1	M	70	Yes	2022	MHE	Dec 2022
P2	F	76	No	<2009	HE gr. 1	Feb 2023
P3	F	63	Yes	2020	None/MHE	Jan 2023
P4	F	69	No	2019	None/MHE	Dec 2022
**Participant HCP**				**Gender** **(M/F)**	**Years Since Education/Years in Specialty**	
HCP1				F	16/8	
HCP2				F	2.5/2	
HCP3				M	18/15	
HCP4				M	10/5	

## Data Availability

Data supporting reported results will be shared upon request by contacting the corresponding authors. Dataset available on request from the authors due to privacy and ethical considerations. Furthermore, all interview transcripts are in Danish.

## Supplementary Materials

The following supporting information can be downloaded at: https://www.mdpi.com/article/10.3390/healthcare14070874/s1, File: Interview guides and Illustration of conclusion.

## Abbreviations

ALD	Alcoholic liver disease
EASL	European Association for the Study of the Liver
HCP	Healthcare professional
HE	Hepatic encephalopathy
HR-QoL	Health-related quality of life
ID	Interpretive description
MASLD	Metabolic-associated steatotic liver disease
MHE	Minimal hepatic encephalopathy
