# Supplementary material for: Becoming a Different Person: Living with Hepatic Encephalopathy as a Condition in Everyday Life—A Qualitative Explorative Study
Source: Healthcare (Basel). 2026 Mar 28;14(7):874. doi: 10.3390/healthcare14070874 (PMC13073394; doi:10.3390/healthcare14070874)
Supplement: Supplementary file 1 [file healthcare-14-00874-s001.zip › healthcare-4162215-supplementary.pdf]

Interview guide for patient interviews

Research questions – Explaines	Interview questions – Describes	Follow-up and probing questions – generic and applicable throughout the interview
**1. Opening Questions:**<br><br>Generally, about the illness/disease and diagnosis<br><br>Experiences and perceptions of living with chronic liver disease – from your own perspective<br><br>Inspiration to see oneself/the situation from the outside - an indirect question	For how long have you been living with liver disease?<br><br>OR<br><br>For how long have you had liver disease?<br><br>Can you tell me what it's like to live with chronic liver disease? / Can you tell me about having chronic liver disease?<br><br>How would your family and friends describe you before and after you received the diagnosis, respectively?	**Follow-up Questions:**<br><br>If you recall the time before you were diagnosed – what did it mean to receive the diagnosis, when did you get it, who gave it to you, what were you told?<br><br>**Probing Questions:**<br><br>Can you tell me more about that specific day/episode…?
**2. Specifically, about the symptom hepatic encephalopathy**	Do you remember when you first experienced brain dysfunction?<br><br>Try to describe/tell me how you experienced it/what happened?	Have you experienced/tried it many times? Is it the same each time? How does it differ?<br><br>Ask about physical sensations as well as mental, emotional, and practical experiences – what happens exactly?

**3. Everyday life experiences with hepatic encephalopathy**<br><br>• Social life<br>• Work<br>• At home<br>• Outside home<br>• Activities<br>• Sleep<br>• Well-being	How has your life been affected by the brain dysfunction? / the cognitive impairment?<br><br>Do you have any thoughts on how your surroundings (depending on who is closest) experience it?	**Indirect, probing:**<br><br>Do you talk about it? Do you talk to others about it (Identity, relations, work life, spare time, driving e.g.)?
Time between episodes (relating to the symptom being recurrent)	Would you try to describe a typical day in your life when you are not experiencing brain dysfunction/cognitively impaired?<br><br>How do you solve specific challenges?<br><br>Do you have any memory aids or tools that help you in your daily life?	Do you take any special precautions during the periods of time when you are not cognitively impaired – if so, which ones?<br><br>What is particularly challenging for you?<br><br>Are there any activities you have stopped due to your illness?<br><br>Does it make you feel worried in your daily life?
The condition of HE being a recurrent symptom	What do you think about the possibility of experiencing cognitive impairment/brain dysfunction again?	Insecurity, anxiety, precautions, thoughts about the future…

**4. Encounters with the healthcare system and the experience of having one’s needs met**	Try to describe your most recent hospitalization due to brain dysfunction?<br><br>Can you describe what it is like to attend the outpatient clinic?<br><br>What is most important to you when you visit the outpatient clinic?<br><br>Do you feel you can be honest with the health care professionals?<br><br>Do you get help with the problems you have/Do you get the help you need?	What do you remember?<br><br>How did you arrive, who was with you?<br><br>Where, how, and by whom were you received?<br><br>What were you told?<br><br>What do you talk about? Do you get answers to your questions?<br><br>E.g. time-specific, continuity in seeing the same nurse, help with remembering medication, information (new/repeated)...<br><br>**Probing questions:** Ask about a specific visit, e.g., the most recent one.<br><br>Is there anything you feel is missing in the interveiw – or something you are particularly satisfied with?

**5. Closing**	At the end, is there anything we haven't talked about that you think is important for me to know – or for you to share?<br><br>How did you experience the interview?<br><br>May we contact you by phone later if we have any follow-up questions?	**THANK YOU VERY MUCH!**

The interview guide was developed with inspiration from

Tanggaard & Brinkmann (2020) [25] and *Green & Thorogood (2018)* [26]

Supplementary:

The overall synthesis of the findings (figure below) shows both static conditions (square in the middle: the symptom, the diagnosis, alcohol, and stigmatization) as well as an opportunity space for the clinical encounter, which can be influenced and modified in local clinical practice. The underlying vulnerability can be met through the relationship between patient and HCPs.

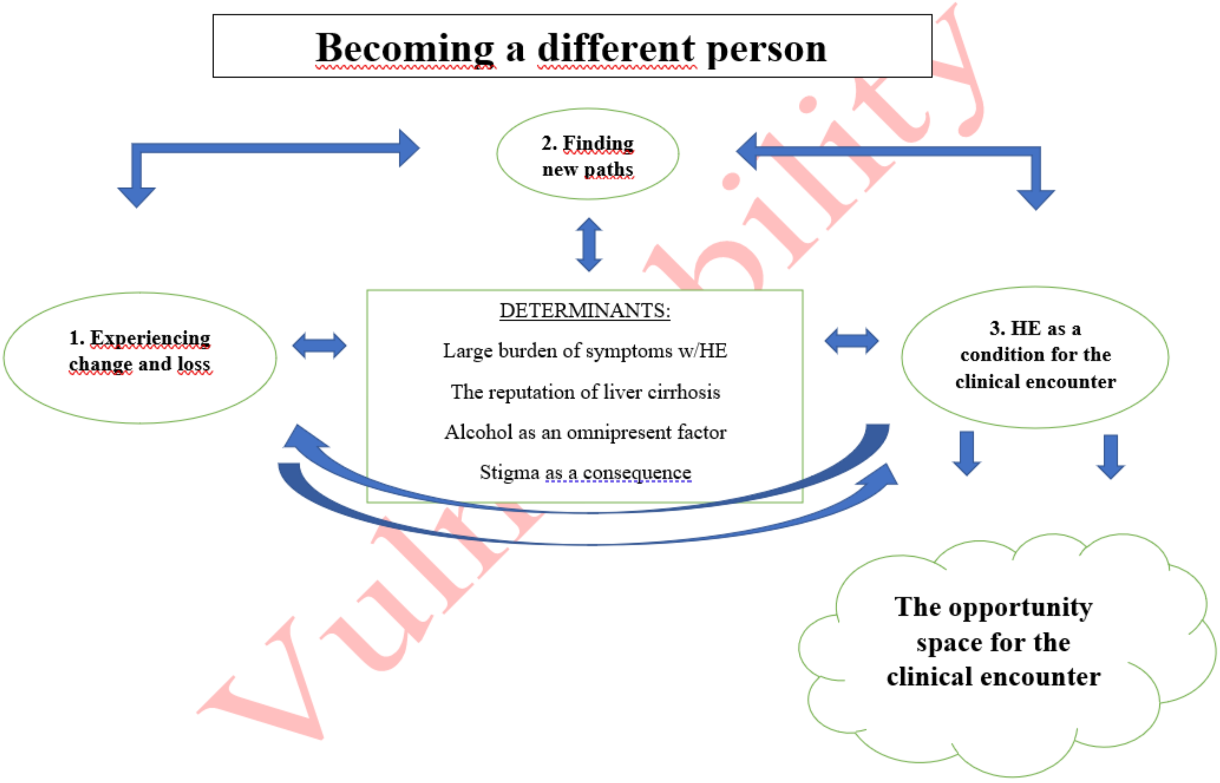

Figure: Conclusion
